# Temporal trends in occupational injuries treated in US emergency departments, 2012–2019

**DOI:** 10.1186/s40621-023-00423-y

**Published:** 2023-03-10

**Authors:** Eric W. Lundstrom, Scott A. Hendricks, Suzanne M. Marsh, Caroline P. Groth, Gordon S. Smith, Ruchi Bhandari

**Affiliations:** 1 Department of Epidemiology and Biostatistics, School of Public Health, West Virginia University, 64 Medical Center Dr, Morgantown, WV 26506 USA; 2 Division of Safety Research, National Institute for Occupational Safety and Health, Morgantown, WV USA

**Keywords:** Occupational injuries, Time-series analysis, Seasonality, Emergency departments, Injury surveillance

## Abstract

**Background:**

Evidence suggests that rates of occupational injuries in the US are decreasing. As several different occupational injury surveillance systems are used in the US, more detailed investigation of this trend is merited. Furthermore, studies of this decrease remain descriptive and do not use inferential statistics. The aim of this study was to provide both descriptive and inferential statistics of temporal trends of occupational injuries treated in US emergency departments (EDs) for 2012 to 2019.

**Methods:**

Monthly non-fatal occupational injury rates from 2012 to 2019 were estimated using the national electronic injury surveillance system—occupational supplement (NEISS-Work) dataset, a nationally representative sample of ED-treated occupational injuries. Rates were generated for all injuries and by injury event type using monthly full-time worker equivalent (FTE) data from the US Current Population Survey as a denominator. Seasonality indices were used to detect seasonal variation in monthly injury rates. Trend analysis using linear regression adjusted for seasonality was conducted to quantify changes in injury rates from 2012 to 2019.

**Results:**

Occupational injuries occurred at an average rate of 176.2 (95% CI =  ± 30.9) per 10,000 FTE during the study period. Rates were highest in 2012 and declined to their lowest level in 2019. All injury event types occurred at their highest rate in summer months (July or August) apart from falls, slips, and trips, which occurred at their highest rate in January. Trend analyses indicated that total injury rates decreased significantly throughout the study period (− 18.5%; 95% CI =  ± 14.5%). Significant decreases were also detected for injuries associated with contact with foreign object and equipment (− 26.9%; 95% CI =  ± 10.5%), transportation incidents (− 23.2%; 95% CI =  ± 14.7%), and falls, slips, and trips (− 18.1%; 95% CI =  ± 8.9%).

**Conclusions:**

This study supports evidence that occupational injuries treated in US EDs have decreased since 2012. Potential contributors to this decrease include increased workplace mechanization and automation, as well as changing patterns in US employment and health insurance access.

**Supplementary Information:**

The online version contains supplementary material available at 10.1186/s40621-023-00423-y.

## Background

Non-fatal occupational injuries represent a significant source of morbidity for workers in the United States (US), with an estimated 1,108,300 non-fatal occupational injuries requiring time away from work in 2019 (U.S. Bureau of Labor Statistics 2020a). Furthermore, occupational injuries cost the US economy an estimated $171 billion in 2019 alone (National Safety Council 2022). In addition to a large national economic burden, occupational injuries result in significant psychosocial harm to workers (Kim and Choi 2016; Lax and Klein 2008), their families (e.g., through lost earnings and an increased time spent caring for an injured family member; Boden 2005; Dembe 2001), and their communities (Boden et al. 2001).

A crucial step in preventing occupational injuries is epidemiologic surveillance (Azaroff et al. 2002). As the US has no centralized occupational injury reporting system, non-fatal injury surveillance occurs through multiple sources, including emergency department (ED) records, employer-based surveys, and workers compensation claims (National Academy of Science 2018; Bush et al. 2021). Each source has relative strengths and weaknesses. For instance, ED-treated injuries, collected via the National Electronic Injury Surveillance System—Occupational Supplement (NEISS-Work), represent workers of any employment type (e.g., public, private, self-employed, volunteers, etc.) but are limited to workers who seek ED treatment (National Institute for Occupational Safety and Health (NIOSH) Division of Safety Research 2019a). Conversely, employer-reported injury data, collected through the Bureau of Labor Statistics’ (BLS) Survey of Occupational Injuries and Illnesses (SOII), are collected through a large survey with high response rates but are limited to injuries incurred by privately employed workers (Williams 2022; Council of State and Territorial Epidemiologists 2021); while some state and local government employees are included in SOII, injuries incurred by federal and self-employed workers are not captured (Wiatrowski 2014). Finally, workers compensation data include many variables and allow for individual-level longitudinal analysis, but require an injury to be billed to, or have a claim associated with, a worker’s compensation system (Seabury et al. 2014; Witt et al. 2018). Previous literature estimates that over 40% of ED-treated occupational injuries nationally are not billed to workers’ compensation (Groenewold and Baron 2013) and that workers’ compensation is the expected payer in less than 5% of ED-treated occupational injuries at the state level (Bush et al. 2021). Furthermore, workers’ compensation datasets are typically available only at the state level or for small proportions of the national working population (Murphy et al. 2021).

Despite their differences, several independent data sources report decreases in US non-fatal occupational injury rates, continuing a decades-long trend of decline (Bhushan and Leigh 2011). For instance, Guerin et al. reported that annual occupational injury rates treated in US EDs declined from 2012 to 2018 for workers aged 18–44 years (Guerin et al. 2020). Similarly, employer-reported data from the BLS SOII indicate that non-fatal occupational injuries and illnesses decreased from 3.7 per 100 full-time worker equivalents (FTE) in 2012 to 3.0 in 2019 (U.S. Bureau of Labor Statistics 2013; U.S. Bureau of Labor Statistics 2020b). Previous studies have suggested that several factors may potentially be contributing to these declines, including the outsourcing of dangerous jobs to lower-income countries (Abdalla et al. 2017), increased mechanization (Issa et al. 2019), and the implementation of targeted safety regulations (Monforton and Windsor 2010). Additionally, several factors may affect occupational injury surveillance without changing the rate at which workers incur injuries, such as decreased injury reporting as a result of changing rates of unionization (Morse et al. 2003) or changes to health insurance access (Berdahl and Zodet 2010).

Although data suggest US occupational injury rates are declining, current literature describing trends in US all-industry occupational injuries is limited to annual descriptive statistics; inferential times-series analyses of national injury trends have largely been used only to assess the impact of safety interventions within single industries (Monforton and Windsor 2010) or trends in specific types of occupational injuries (e.g., non-fatal traumatic brain injuries; Konda et al., 2015). Likewise, studies using US occupational injury surveillance data regularly exclude the assessment of seasonality, a temporal pattern common in injury data. Thus, we aimed to use NEISS-Work, a nationally representative database of occupational injuries treated in US EDs, to assess temporal trends in ED-treated occupational injuries in the US from 2012 to 2019. The specific aims of this study were: (1) to report yearly national injury rate estimates, both overall and by injury event type, (2) to report seasonality of monthly injury rate estimates, both overall and by injury event type, and (3) to report inferential statistics on trends in occupational injury rates during the study period.

## Methods

### Data source

Non-fatal occupational injury data for the years 2012 through 2019 were obtained from NEISS-Work, a nationally representative database of non-fatal occupational injuries treated in US EDs. The National Institute for Occupational Safety and Health (NIOSH) obtains the data for NEISS-Work through an inter-agency agreement with the Consumer Product Safety Commission (CPSC), the agency responsible for collecting the NEISS-Work data. For the purposes of NEISS-Work, an occupational injury is defined as an injury for which an ED chart or other hospital record indicates that the injury involved a non-institutionalized civilian who was injured while working for pay or compensation of any kind, working on a farm, or volunteering for an organization (Marsh et al. 2016; Reichard and Marsh 2021).

The NEISS-Work data are collected through a probability sample of approximately 67 hospitals that report non-fatal data on occupational injuries seen in their EDs to the CPSC via coders trained to identify the work relatedness of occupational injury data based on extensive manual review of hospital admission information and ED chart inspection. NEISS-Work does not rely on International Statistical Classification of Diseases (ICD) codes or workers compensation billing status (National Institute for Occupational Safety and Health (NIOSH) Division of Safety Research 2021a) to identify cases, although the latter may be used as part of the overall manual chart review case identification process. Participating hospitals are stratified based on annual number of ED visits. Hospitals must have a minimum of six beds and a 24-h ED for inclusion. Individual cases reported to NEISS-Work are weighted based on the inverse probability of the reporting hospital being included in the sample so that the estimates represent population total injuries for the US (National Institute for Occupational Safety and Health (NIOSH) Division of Safety Research 2021a).

Data for 2012–2019 were chosen as this was the longest period for which data for injury event were all comparably coded to the same version (v 2.01) of the BLS Occupational Injury and Illness Classification System (OIICS). BLS OIICS codes are used to assign injury event and diagnosis codes in NEISS-Work using a narrative comment field developed by coders through review of ED chart and hospital admission data. Data for years prior to 2012 were coded based on the BLS OIICS v 1.01 (National Institute for Occupational Safety and Health (NIOSH) Division of Safety Research 2021b). The shift from the BLS OIICS v 1.01 to v 2.01 in 2012 was considered a break in series. Furthermore, the 2019 data were the most recent data available at the time of analysis (National Institute for Occupational Safety and Health (NIOSH) Division of Safety Research 2021a). Due to a series break that resulted in the exclusion of most illness cases starting with data from 2015, data for 2012–2014 were re-reviewed to ensure compatibility throughout the study period (National Institute for Occupational Safety and Health (NIOSH) Division of Safety Research 2021a).

### Statistical analysis

All data were stored on a secure drive accessible only to the study team. Statistical analyses were performed in Rstudio version 4.0.1 (Rstudio Team 2022). Using the NEISS-Work dataset, national ED-treated occupational injury count estimates were produced using the R packages “survey” and “srvyr” (Ellis et al. 2021; Lumley 2021) using the aforementioned NEISS-Work survey weights. ED-treated occupational injury count estimates were generated for all injuries and by injury event type, a categorical variable denoting the way an injury was incurred and is based on the aforementioned BLS OIICS v 2.01 classification system (National Institute for Occupational Safety and Health (NIOSH) Division of Safety Research 2021b); all analyses were conducted both for total injury rate estimates and stratified by injury event type. ED-treated occupational injury rates were calculated per 10,000 FTE using Current Population Survey (CPS) estimates which were generated using NIOSH’s Employed Labor Force (ELF) query system; as NEISS-Work includes all work-related ED-treated injuries, FTE estimates were generated for all jobs (as opposed to “primary” or “secondary” jobs only) (National Institute for Occupational Safety and Health (NIOSH) Division of Safety Research 2021c). Standard errors (SE) for FTE estimates were generated using generalized variance functions provided by BLS; standard errors were used to calculate monthly FTE variances by multiplying the square of the SE by corresponding ELF-generated monthly FTE estimates (i.e., the corresponding monthly sample size) (National Institute for Occupational Safety and Health (NIOSH) Division of Safety Research 2021c). Variances of both numerator (injury count estimates) and denominator (FTE) data were used to calculate 95% confidence intervals (CI) for ED-treated occupational injury rate estimates based on Taylor series expansion (National Institute for Occupational Safety and Health (NIOSH) Division of Safety Research 2021d) and were reported as injury rate estimates ± margin of error.

Seasonality of injury rate estimates was assessed by calculating seasonality indices per month. Seasonality indices were calculated by dividing the mean rate for each month by the mean monthly occupational injury rate for the entire dataset; seasonality indices of greater and less than one indicate higher than and lower than expected injury rates for a given month, respectively (Zhang et al. 2014).

To assess linear trends in injury rates over time, we fit a linear regression model to monthly injury rate estimates and adjusted for autocorrelation and serially correlated error terms using autoregressive integrated moving average (ARIMA) modeling. This analysis was conducted using both monthly total injury rate estimates and monthly estimates stratified by injury event type. In data violating the linear regression assumption of no autocorrelation, ARIMA models are used to control for serial correlation (e.g., seasonality) by including lagged dependent variable values and errors, including in studies of injury data (Box et al. 2016; Zhu et al. 2015). An ARIMA model takes the form ARIMA(p,d,q)(P,D,Q)_m_, where *p* is the order of autocorrelation, *d* is the number of differences applied to the data, *q* is the order of moving average terms, *P, D,* and *Q* are the seasonal versions of these terms, and *m* is the order of seasonality (e.g., 12 for annually seasonality in monthly data) (Hyndman and Athanasopoulos 2018a). ARIMA models were fit to monthly injury rates by examining autocorrelation and partial autocorrelation plots. A lagged regression estimate was included if it showed statistical significance (*p* < 0.05) and was necessary to control for serial correlation. Finally, significance of each model’s Ljung-Box *Q* statistic was observed to ensure proper model fit, with a non-significant value considered a properly fit model (Ljung and Box 1978). The conditional sum of squares method was used to estimate all models. To assess temporal trends, a trend regressor with slope of one was included in each ARIMA model as a covariate and reported with 95% CIs (Hyndman and Athanasopoulos 2018b). A total percent decrease in injury rates throughout the study period was estimated by multiplying this term by 96 (i.e., the total number of months in the study period) and calculating the percent difference from the model’s intercept; an analogous calculation using each trend parameter’s 95% CI was performed to determine each percent decrease’s 95% CI.

## Results

**Fig. 1:**
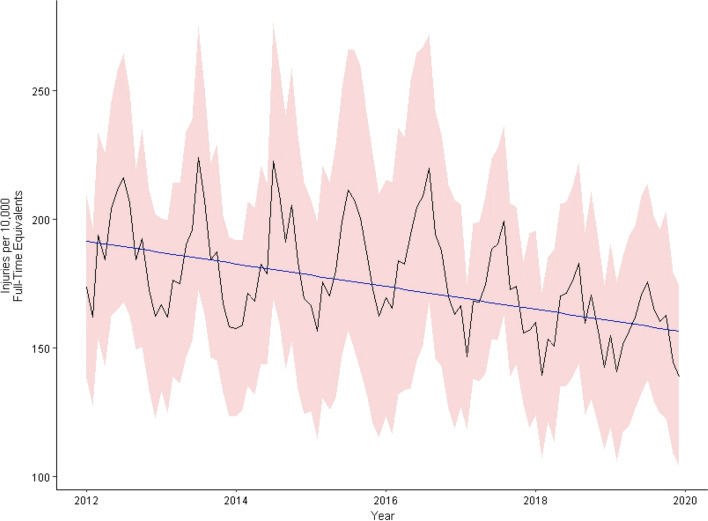
Monthly injury rate estimates for occupational injuries treated in US EDs, 2012–2019. Numerator data (monthly ED-treated injury count estimates) are from the National Emergency Injury Surveillance System—Occupational Supplement (NEISS-Work) dataset and were produced using the R packages “survey” and “srvyr.” Denominator data (FTE) were obtained from the Current Population Survey (CPS) via the NIOSH Employed Labor Force querying system. Variances from both numerator and denominator data were used to calculate for injury rate 95% CI (represented here by red shading) using a Taylor series expansion. Blue line represents a linear trend parameter adjusted for seasonality using ARIMA modeling

Monthly estimates of occupational injuries treated in the US EDs with 95% confidence intervals are presented graphically in Fig. 1 (graphical representation of monthly estimates of injury rates by injury event type is available in Additional files 1, 2, 3, 4, 5, 6: Figs. S1–S6). Injuries were incurred at an average rate of 176.2 (95% CI =  ± 30.9) per 10,000 FTE during the study period (Table 1). Annual injuries were estimated at their highest rate in 2012 (188.4 ± 38.9 per 10,000 FTE) and their lowest in 2019 (156.8 ± 34.5 per 10,000 FTE). Injuries caused by contact with objects and equipment had the highest cause-specific rate during the study period (58.6 ± 0.4 per 10,000 FTE); followed by overexertion and other bodily reactions (48.5; ± 10.6 per 10,000 FTE); falls, slips, and trips (27.7 ± 4.8 per 10,000 FTE); exposure to harmful substances or environments (17.9 ± 3.9 per 10,000 FTE); and violence and other injuries by persons or animals (15.5 ± 3.5 per 10,000 FTE). Analyses of rates of monthly injuries caused by fires and explosions, as well as nonclassifiable sources, were not reported due to NEISS-Work sample size reporting standards (unreliably small numbers).

**Table 1:** Mean annual occupational injuries (per 10,000 FTE) treated in US emergency departments, by injury event type^a^

Year	All injuries	Violence and other injuries by persons or animals	Transportation incidents	Falls, slips, and trips	Exposure to harmful substances or environments	Contact with objects and equipment	Overexertion and other bodily reaction
2012	188.4 ± 38.9	15.6 ± 4.7	6.4 ± 1.5	29.2 ± 6.0	17.9 ± 3.7	65.9 ± 14.0	49.6 ± 11.8
2013	182.6 ± 38.4	15.9 ± 4.8	5.2 ± 1.2	28.8 ± 5.6	17.9 ± 3.9	64.8 ± 14.4	48.0 ± 11.9
2014	183.1 ± 35.6	15.3 ± 3.9	5.4 ± 1.2	29.8 ± 5.8	17.3 ± 3.9	62.0 ± 12.8	50.4 ± 11.6
2015	182.1 ± 48.4	16.1 ± 4.3	5.3 ± 1.4	29.6 ± 8.3	18.9 ± 6.2	61.3 ± 16.0	48.9 ± 14.9
2016	186.8 ± 47.8	16.7 ± 4.4	5.2 ± 1.2	29.1 ± 7.9	19.3 ± 6.1	58.9 ± 14.3	55.0 ± 17.1
2017	171.5 ± 31.2	16.1 ± 3.6	5.1 ± 1	25.4 ± 4.6	18.7 ± 4.4	55.4 ± 10.1	47.6 ± 10.8
2018	160.9 ± 34.2	14.3 ± 3.5	4.4 ± 1	24.8 ± 5.1	17.3 ± 5.0	51.8 ± 10.0	45.8 ± 12.5
2019	156.8 ± 34.5	14.6 ± 3.3	5.0 ± 1.1	25.3 ± 5.4	16.1 ± 4.4	50.4 ± 10.5	43.1 ± 12.4
Total	176.2 ± 30.9	15.5 ± 3.5	5.2 ± 0.9	27.7 ± 4.8	17.9 ± 3.9	58.6 ± 10.4	48.5 ± 10.6

^a^Numerator data (monthly ED-treated injury count estimates) are from the National Electronic Injury Surveillance System—Occupational Supplement (NEISS-Work) dataset and were produced using the R packages “survey” and “srvyr.” Denominator data (FTE) were obtained from the Current Population Survey (CPS) via the NIOSH Employed Labor Force query system. Variances from both numerator and denominator data were used to calculate 95% CIs using a Taylor series expansion, which is reported as each injury rate estimate ± margin of error. Injury event type definitions are based on the Bureau of Labor Statistics Occupational Injury and Illness Classification System, version 2.01

Rates varied widely by month and seasonality indices for total injury rates were greatest in July (1.15) and lowest in February (0.87) (Table 2). With the exception of falls, slips, and trips, all other injury event types showed similar seasonality (lowest seasonality index in February, highest in July or August), including injuries caused by violence (February = 0.82; July = 1.18), transportation incidents (February = 0.87; July = 1.18), exposure to harmful substances (February = 0.81; August = 1.45), and overexertion (February = 0.86; August = 1.10). Falls, slips, and trips were the only injury event type to have greatest seasonality index in a winter month with highest and second highest seasonality indices occurring in January (1.17) and February (1.16), respectively; a second peak in falls, slips, and trips occurred the summer (July = 1.04; August = 1.03). Injuries caused by falls, slips, and trips occurred at their lowest rate in April with a seasonality index of 0.85.

**Table 2:** Seasonality indices^a^ of occupational injuries (per 10,000 FTE) treated in US emergency departments by injury event type^b^

Month	All injuries	Violence and other injuries by persons or animals	Transportation incidents	Falls, slips, and trips	Exposure to harmful substances or environments	Contact with objects and equipment	Overexertion and other bodily reaction
January	0.93	0.87	0.94	1.17	0.82	0.88	0.91
February	0.87	0.82	0.87	1.09	0.81	0.81	0.86
March	0.97	0.95	0.93	1.00	0.87	0.96	1.01
April	0.96	1.02	0.92	0.85	0.89	0.96	1.02
May	1.03	1.08	1.02	0.97	1.03	1.06	1.02
June	1.07	1.06	1.10	0.98	1.15	1.13	1.04
July	1.15	1.18	1.15	1.04	1.45	1.16	1.08
August	1.13	1.10	1.03	1.03	1.31	1.17	1.10
September	1.02	1.06	1.06	0.95	1.05	1.05	1.01
October	1.04	1.04	1.04	0.99	0.94	1.08	1.05
November	0.94	0.94	0.99	0.94	0.88	0.91	0.97
December	0.89	0.88	0.94	0.99	0.82	0.84	0.92

^a^Calculated by dividing the mean rate for each month by the mean monthly occupational injury rate for the entire dataset

^b^Numerator data (monthly ED-treated injury count estimates) are from the National Emergency Injury Surveillance System—Occupational Supplement (NEISS-Work) dataset and were produced using the R packages “survey” and “srvyr.” Denominator data (FTE) were obtained from the Current Population Survey (CPS) via NIOSH Employed Labor Force querying system. Injury event type definitions are based on the Bureau of Labor Statistics Occupational Injury and Illness Classification System version 2.01

Table 3 presents trend analysis of injury rate estimates, both by month and by month and injury event type, as well as the ARIMA structure used to control for serial data correlation (e.g., seasonality) in each model. Total injury rates in January 2012 were estimated to be 191.8 per 10,000 FTE, as denoted by the model’s intercept. Total injury rate estimates decreased at a rate of − 0.37 (95% CI =  ± 0.29) per month and were estimated to be 156.3 per 10,000 FTE by the end of the study period (December 2019), resulting in an overall decrease of 18.5% (95% CI =  ± 14.5%). Stratifying the data by month and injury event type, significant decreases were detected in monthly rates of injuries associated with contact with foreign objects and equipment (− 26.9%; 95% CI =  ± 10.5%); transportation incidents (− 23.2%; 95% CI =  ± 14.7%); and falls, slips, and trips (− 18.1%; 95% CI =  ± 8.9%). Monthly rates of injuries for some injury event types, including those associated with violence; exposure to harmful substances; and overexertion and bodily reaction, showed non-significant decreases.

**Table 3:** Trend analysis of monthly ED-treated occupational injury rates estimates per 10,000, 2012–2019^a^

Injury type	ARIMA structure^b^	Intercept	Trend parameter (± 95% CI)	Percent decrease, 2012–2019 (± 95% CI)^c^
All injuries	(1,0,2)(1,0,0)_12_	191.8	**− 0.37 (± 0.29)**	**− 18.5% (± 14.5%)**
Violence	(1,0,0)(1,0,0)_12_	15.9	**− **0.01 (± 0.03)	**− **6.2% (± 14.9%)
Transportation incidents	(1,0,0)(0,0,0)_12_	5.9	**− 0.01 (± 0.01)**	**− 23.2% (± 14.7%)**
Falls, slips, and trips	(0,0,0)(1,0,0)_12_	30.9	**− 0.06 (± 0.03)**	**− 18.1% (± 8.9%)**
Exposure to harmful substances	(1,0,0)(1,0,0)_12_	18.4	**− **0.02 (± 0.05)	**− **9.3% (± 24.9%)
Contact with foreign objects and equipment	(1,0,3)(1,0,1)_12_	68.1	**− 0.19 (± 0.08)**	**− 26.9% (± 10.5%)**
Overexertion and bodily reaction	(1,0,0)(1,0,0)_12_	51.3	**− **0.06 (± 0.09)	**− **12.6% (± 16.3%)

Significant values bolded

^a^Numerator data (monthly ED-treated injury count estimates) are from the National Emergency Injury Surveillance System—Occupational Supplement (NEISS-Work) dataset and were produced using the R packages “survey” and “srvyr.” Denominator data (FTE) were obtained from the Current Population Survey (CPS) via NIOSH Employed Labor Force querying system. Injury event type definitions are based on the Bureau of Labor Statistics Occupational Injury and Illness Classification System version 2.01

^b^An ARIMA(p,d,q)(P,D,Q)_m_ structure was used to control for serial correlation (e.g., seasonality) in monthly injury rate data, where p is the order of autocorrelation, d is the number of differences applied to the data, q is the order of moving average terms, P,D, and Q are the seasonal versions of these terms, and m is the order of seasonality (e.g., 12 for annually seasonality in monthly data). A linear trend parameter was used to measure overall decreases

^c^Calculated by multiplying each model’s trend parameter and 95% CI by 96 (i.e., the total number of months in the study period) and calculating percent difference from the model’s intercept; significant decreases are bolded

## Discussion

Using the NEISS-Work dataset, one of the primary workplace injury surveillance programs in the US, we analyzed rates of occupational injuries treated in US EDs from 2012 to 2019. We found that injury rates during the study period were greatest in 2012 (188.4 ± 38.9 per 10,000 FTE) and lowest in 2019 (156.8 ± 34.5 per 10,000). ED-treated injuries displayed a marked seasonal pattern, with seasonality indices at their greatest in summer months (July or August) and lowest during winter months (December, January, or February). Seasonality indices for rates stratified by injury event type followed a similar pattern, apart from falls, slips, and trips, which had a peak seasonality index in January. Additionally, we observed a decrease in estimated rates of occupational injuries treated in US EDs of 18.5% (95% =  ± 14.5%) throughout the study period.

The BLS SOII, another major US occupational injury surveillance program, also reported a decrease in occupational injury rates throughout our study period. However, SOII recorded annual injury rates of 3.7 and 3.0 per 100 FTE for 2012 and 2019, respectively, nearly double the rates estimated in our study for those years (U.S. Bureau of Labor Statistics 2013; U.S. Bureau of Labor Statistics 2020b) (Table 1). A discrepancy in occupational injury rates between these two datasets has been noted in previous literature and is likely because NEISS-Work primarily captures injuries severe enough to require ED treatment, a fraction of the total number of injuries incurred in the US (Chen 2009). In contrast, SOII captures any injury in its sample reported by an employer in accordance with OSHA recordkeeping guidelines (National Academy of Sciences 2018; Council of State and Territorial Epidemiologists 2021). As NEISS-Work and SOII have different mechanisms for capturing injuries, the fact that they both display a decrease from 2012 to 2019 strengthens evidence that US non-fatal occupational injury rates have decreased during this period.

To the authors’ knowledge, no study has used workers’ compensation data to estimate trends in national occupational injury rates throughout our study period; this is expected as the US does not have a national workers’ compensation system. However, state-level workers’ compensation studies, such as one study from Ohio for 2007–2017, also note state-wide decreases in injury rates throughout our study period (Wurzelbacher et al. 2021). Additionally, previous literature has noted differences in occupational injury rate estimates generated via ED-based and workers’ compensation data, with one study finding that occupational concussion injury rates in Kentucky measured via ED data (21.7 per 100,000 employed civilians) were higher than those reported by workers compensation (11.7 per 100,000; Slavova and Bunn 2015). This same study found that the estimated rate of injuries was highest when using linked ED, hospital discharge, and workers’ compensation data (31.8 per 100,000), implying that each surveillance system has inherent strengths in capturing occupational injuries.

We noted a seasonal pattern in which injury rate estimates were greatest in a summer month (July or August) and lowest in a winter month (December, January, or February) which has been attributed in other studies to increased heat and humidity, as well as an influx of temporary workers and increased construction during summer months (Oleske and Hahn 1992; Taylor et al. 2002). A similar pattern of seasonality has been noted in previous occupational injury literature. For example, Peirce calculated seasonality indices of occupational injury rates using 2003–2010 SOII data and found that injuries peaked in seasonality in July at an index of 1.12, similar to our peak index of 1.15 in the same month for total injury rates (Pierce 2013). However, Peirce’s indices were lowest in December (seasonality index = 0.86) compared to February (seasonality index = 0.87) in our study, which they suggest may be influenced by lower end-of-year reporting in SOII. Categorized by injury event type, injury rate estimates in our study followed a similar seasonality pattern except for falls, slips, and trips, which peaked in January (seasonality index = 1.17). An increased rate of fall and slip injuries in winter months, or in association with cold weather, has been noted in previous literature. For example, studies of the mining industry have found an inverse relationship between temperature and incidence of fall and slip injuries (Bell et al. 2000; Hassi et al. 2000). This association is likely influenced by workers’ frequent contact with snow or icy surfaces during winter months (Chang et al. 2016), a hypothesis supported by Bentley and Haslam’s finding that the majority of slip injuries in a sample of mail delivery workers involved snow or ice (Bentley and Haslam 2001). Furthermore, survey data from Bentley and Haslam’s study indicate that 90% of mail delivery workers consider contact with slick surfaces to be a major contributing factor to occupational fall and slip injuries.

Several factors have likely influenced recent declines in US occupational injury rates, including reducing hazardous jobs and increased safety practices. Studies suggest that ergonomic interventions (Fathallah et al. 2008; National Research Council and Institute of Medicine Panel on Musculoskeletal Disorders and the Workplace 2001) and increasingly mechanized workplaces (Issa et al. 2019) have resulted in fewer jobsite hazards. One example of such a shift is within the logging industry, which regularly experiences injury rates far beyond the US all-industry average (Janocha and Hopler 2018; Myers et al. 1998). As this industry has seen the introduction of mechanized timber harvesting in recent decades, studies show that logging companies have experienced significant decreases in injury rates after transitioning from manual (i.e., non-mechanized, chainsaw-based) to mechanized timber harvesting (Bell 2002). Similarly, increases in occupational automation have further removed workers from the physical production process and made several workplaces safer (Autor 2015; Leso et al. 2018). In fact, one study found that for every standard deviation increase in workplace automation, occupational injuries decrease 1.2 per 100 workers (Gihleb et al. 2022). Another potential contributor to decreasing US occupational injury rates is increased globalization (Hämäläinen 2009), defined within an occupational health context as “…the transfer of manufacturing from Established Economic Markets (US and European Community as defined by the World Bank) to ‘developing’ economic markets” (Schulze 2007). As laborious, high-risk manufacturing jobs are transferred to developing nations, an unintended consequence is that workers in higher income countries must find lower-risk employment (Abdalla et al. 2017). This can be seen in changing US manufacturing industry employment rates, which decreased 4.5% from 2012 to 2019 (the period analyzed in this study) (U.S. Bureau of Labor Statistics 2022a). Employment rates in some other goods-producing sectors, which have higher rates of occupational injuries relative to other sectors (U.S. Bureau of Labor Statistics 2020b), have also decreased (e.g., logging and mining employment rates decreased 31.2% for 2012–2019).

These and other employment trends may have influenced our stratified analysis, which noted significant decreases in the rate of injuries associated with certain injury event types but not others (Table 3). For example, injuries due to contact with foreign objects and equipment decreased 26.9% during our study period, more than any other injury event type. Nationally, approximately 20% of occupational injuries due to contact with objects and equipment are incurred in the manufacturing industry (National Safety Council 2023); as noted previously, manufacturing employment rates decreased throughout our study period (U.S. Bureau of Labor Statistics 2022a). In contrast, violence injuries decreased at the lowest rate of any injury event type throughout our study period and this decrease was not significant (− 6.2% (± 14.9%)). As the majority (76%) of workplace violence injuries requiring days away from work are incurred by workers in the health care and social assistance industries (National Institute for Occupational Safety and Health 2022), this finding may have been influenced by increasing employment in these industries throughout our study period (12.9–13.5% from 2012 to 2019, respectively) (U.S. Bureau of Labor Statistics 2022b). While these examples represent plausible associations, we cannot definitively conclude a relationship between employment in a single industry and the trends reported in our study as NEISS-Work did not include detailed industry information for the entire study period.

Some factors may have affected the proportion of occupational injuries captured by the ED-based NEISS-Work without influencing the actual number of injuries incurred by US workers. For example, the annual number of self-employed workers increased 6.4% during our study period (U.S. Bureau of Labor Statistics 2020c). US self-employed workers have been noted to have an increased risk of occupational injury (Bunn et al. 2006) yet are not required to have health insurance or workers compensation benefits which may make them less likely to seek medical care; data suggest that the proportion of US self-employed workers lacking health insurance increased throughout our study period (Rothwell and Harlan 2019). Moreover, evidence suggests that NEISS-Work underestimates the number of occupational injuries incurred by self-employed US workers, possibly because they lack health insurance (Bhandari et al. 2016). It may also be the case that more injured workers over time are seeking treatment in non-ED settings. There was an increase of more than 37% in the number of urgent care centers in the US from 2013 to 2019 (Urgent Care Association 2019), which offer significantly less-expensive treatment than US EDs (Ho et al. 2017). Thus, workers lacking access to health insurance and workers compensation may seek care in urgent care centers for minor and non-life-threatening injuries; workers may also be seeking urgent care as opposed to ED treatment given the latter’s convenience and significantly longer wait times (Khairat et al. 2021). Finally, decreasing unionization rates may have had an influence on occupational injury reporting; data from the US Bureau of Labor Statistics show that the total, all-industry unionization rate decreased from 11.3 to 10.3% throughout the study period. Previous literature suggests differential reporting of injuries by union status, with non-unionized workers being less likely to report (Altassan et al. 2018; Morse et al. 2003; Robinson and Smallman 2006). Extant literature also indicates that non-unionized workers are less likely to have health insurance than those that are unionized (U.S. Bureau of Labor Statistics, 2019) and may therefore be less likely to seek treatment than unionized workers.

This study has several strengths. One strength is that it examines all ED-treated injuries, not just those required to be reported to the BLS. The NEISS-Work dataset captures occupational injury data regardless of industry and its definition of work includes the self-employed and farm workers, giving it a wider capture of work-related injuries compared to employer-reported datasets, such as the BLS SOII. Additionally, NEISS-Work does not require an injury to be billed to workers’ compensation to be included. This is a crucial strength of this dataset as a large proportion of ED-treated occupational injuries are not billed to workers’ compensation (Groenewold and Baron 2013). Finally, to the authors’ knowledge, this is the first study to use inferential time-series techniques to quantify trends in national, all-industry monthly occupational injury data in the US for the period assessed. Specifically, ARIMA modeling, which allows for the analysis of monthly occupational injury data, is an improvement over previous methods used to measure trends in national ED-treated injury data, such as negative binomial regression (Tiesman et al. 2018), which generally cannot account for seasonality. However, other studies have used extensions of ARIMA modeling, such as interrupted time-series (ITS) analysis, to assess the impact of occupational safety and health, such as US Mine Safety and Health Administration regulations (Monforton and Windsor 2010), drugfree workplace interventions (Wickizer et al. 2004), and the influence of a crash prevention program in a large law enforcement agency (Tiesman et al. 2019); ITS analysis may allow future studies to assess the impact of interventions with potential to influence national ED-treated occupational injury rates (e.g., implementation of occupational health and safety policies, changes in workers’ access to health insurance, etc.) were one to be identified.

This study also has several inherent limitations. First, NEISS-Work collects occupational injury data using a probability-based survey sample design. Thus, national occupational injury estimates generated using NEISS-Work are based on a subset of US hospital EDs and include sampling error. ARIMA modeling assumes homoscedasticity of sample variances and is incapable of incorporating any error intrinsic to the NEISS-Work sampling design; incorporating survey design error within our ARIMA model, if possible, would likely increase the width of the confidence intervals presented in Table 3. Despite this, sample variances of injury rate estimates were generally comparable across the study period (Fig. 1), suggesting this limitation likely did not compromise the internal validity of study findings. Second, NEISS-Work only captures injuries treated in a subset of US EDs and do not reflect any change in injury rates due to injuries treated in any other setting. Third, these findings should be discussed only in reference to national, all-industry occupational injury rates, not in any subnational or industry-specific context. Finally, these data do not indicate the severity of the injuries included in NEISS-Work and it is possible that many of the injuries included for analysis were relatively minor; the literature indicates that nearly 90% of US ED-treated injuries are not severe (Villaveces et al. 2013) and most injuries reported to NEISS-Work do not require hospital admission (Konda et al. 2015; Lipscomb et al. 2010; Reichard et al. 2015). As NEISS-Work contains data on whether a patient was hospitalized/transferred after treatment (National Institute for Occupational Safety and Health (NIOSH) Division of Safety Research 2021a), future studies should investigate if hospitalization rates of US ED-treated occupational injuries have changed in recent years. Additionally, as was reported, rates of injuries decreased significantly for some injury event types and not others. Thus, future research should also investigate factors potentially influencing these findings, including injury rate trends by industry and demographic factors.

## Conclusion

To our knowledge, this is the first study to assess temporal trends in a nationally representative dataset of occupational injuries treated in US EDs from 2012 to 2019. We found that annual injury rate estimates were greatest in 2012 and lowest in 2019. Additionally, we provided quantifiable measures of trends in occupational injuries during the study period; previously, only descriptive annual statistics were available to assess trends in such data. Future research should assess the influence of potential mechanisms, such as injury underreporting or shifts in employment, that may have contributed to the trends observed in this study.

## Supplementary Information

**Additional file 1**: **Fig. S1**. Numerator data (monthly ED-treated injury count estimates associated with violence and other injuries by persons or animals) are from the National Emergency Injury Surveillance System—Occupational Supplement (NEISS-Work) dataset and were produced using the R packages “survey” and “srvyr.” Denominator data (FTE) were obtained from the Current Population Survey (CPS) via the NIOSH Employed Labor Force querying system. Variances from both numerator and denominator data were used to calculate for injury rate 95% CI using a Taylor series expansion.

**Additional file 2**: **Fig. S2**. Numerator data (monthly ED-treated transportation injury count estimates) are from the National Emergency Injury Surveillance System—Occupational Supplement (NEISS-Work) dataset and were produced using the R packages “survey” and “srvyr.” Denominator data (FTE) were obtained from the Current Population Survey (CPS) via the NIOSH Employed Labor Force querying system. Variances from both numerator and denominator data were used to calculate for injury rate 95% CI using a Taylor series expansion.

**Additional file 3**: **Fig. S3**. Numerator data (monthly ED-treated falls, slips, and trips injury count estimates) are from the National Emergency Injury Surveillance System—Occupational Supplement (NEISS-Work) dataset and were produced using the R packages “survey” and “srvyr.” Denominator data (FTE) were obtained from the Current Population Survey (CPS) via the NIOSH Employed Labor Force querying system. Variances from both numerator and denominator data were used to calculate for injury rate 95% CI using a Taylor series expansion.

**Additional file 4**: **Fig. S4**. Numerator data (monthly ED-treated injury count estimates associated with exposure to harmful substances or environments) are from the National Emergency Injury Surveillance System—Occupational Supplement (NEISS-Work) dataset and were produced using the R packages “survey” and “srvyr.” Denominator data (FTE) were obtained from the Current Population Survey (CPS) via the NIOSH Employed Labor Force querying system. Variances from both numerator and denominator data were used to calculate for injury rate 95% CI using a Taylor series expansion.

**Additional file 5**: **Fig. S5**. Numerator data (monthly ED-treated injury count estimates associated with contact with objects and equipment) are from the National Emergency Injury Surveillance System—Occupational Supplement (NEISS-Work) dataset and were produced using the R packages “survey” and “srvyr.” Denominator data (FTE) were obtained from the Current Population Survey (CPS) via the NIOSH Employed Labor Force querying system. Variances from both numerator and denominator data were used to calculate for injury rate 95% CI using a Taylor series expansion.

**Additional file 6**: **Fig. S6**. Numerator data (monthly ED-treated injury count estimates associated with overexertion and other bodily reaction) are from the National Emergency Injury Surveillance System—Occupational Supplement (NEISS-Work) dataset and were produced using the R packages “survey” and “srvyr.” Denominator data (FTE) were obtained from the Current Population Survey (CPS) via the NIOSH Employed Labor Force querying system. Variances from both numerator and denominator data were used to calculate for injury rate 95% CI using a Taylor series expansion.

## Data Availability

Data are not publicly available, but limited data can be accessed through the Work-Related Injury Statistics Query System (Work-RISQS) https://wwwn.cdc.gov/wisards/workrisqs/

## Abbreviations

ARIMA: Autoregressive integrated moving average
ED: Emergency department
FTE: Full-time worker equivalent
ITS: Interrupted time series
NEISS-Work: National Electronic Injury Surveillance System—Occupational Supplement
SOII: Survey of Occupational Injuries and Illnesses

## References

[CR1] Abdalla S, Apramian SS, Cantley LF, Cullen MR. Occupation and risk for injuries. Disease control priorities, third edition (volume 7): injury prevention and environmental health. The World Bank; 2017. p. 97–132.

[CR2] Altassan KA, Sakr CJ, Galusha D, Slade MD, Tessier-Sherman B, Cantley LF (2018). Risk of injury by unionization. J Occup Environ Med.

[CR3] Autor DH. Why are there still so many jobs? The history and future of workplace automation. Journal of Economic Perspectives. American Economic Association; 2015. P. 3–30.

[CR4] Azaroff LS, Levenstein C, Wegman DH (2002). Occupational injury and illness surveillance: conceptual filters explain underreporting. Am J Public Health.

[CR5] Bell JL (2002). Changes in logging injury rates associated with use of feller-bunchers in West Virginia. J Safety Res.

[CR6] Bell JL, Gardner LI, Landsittel DP (2000). Slip and fall-related injuries in relation to environmental cold and work location in above-ground coal mining operations. Am J Ind Med.

[CR7] Bentley TA, Haslam RA (2001). Identification of risk factors and countermeasures for slip, trip and fall accidents during the delivery of mail. Appl Ergon.

[CR8] Berdahl TA, Zodet M (2010). Medical care utilization for work-related injuries in the United States 2002–2006. Med Care [internet].

[CR9] Bhandari R, Marsh SM, Reichard AA, Tonozzi TR (2016). Characterizing emergency department patients who reported work-related injuries and illnesses. Am J Ind Med.

[CR10] Bhushan A, Leigh JP (2011). National trends in occupational injuries before and after 1992 and predictors of workers’ compensation costs. Public Health Rep.

[CR11] Boden LI (2005). Running on empty: families, time, and workplace injuries. Am J Public Health.

[CR12] Box GEP, Jenkins GM, Reinsel GC, Ljung GM. Time series analysis: forecasting and control. 5th edn. Wiley; 2016.

[CR13] Bunn T, Costich J, Slavova S (2006). Identification and characterization of Kentucky self-employed occupational injury fatalities using multiple sources, 1995–2004. Am J Ind Med.

[CR14] Bush AM, Bunn TL, Liford M (2021). Identification of work-related injury emergency department visits using international classification of diseases, tenth revision, clinical modification (ICD-10-CM) codes. Inj Prev NLM (medline).

[CR15] Chang W-R, Leclercq S, Lockhart TE, Haslam R (2016). State of science: occupational slips, trips and falls on the same level. Ergonomics [internet].

[CR16] Chen GX (2009). Nonfatal work-related motor vehicle injuries treated in emergency departments in the United States, 1998–2002. Am J Ind Med.

[CR17] Council of State and Territorial Epidemiologists. Occupational health indicators: A guide for tracking occupational health conditions and their determinants [Internet]. 2021. Available from: https://cdn.ymaws.com/www.cste.org/resource/resmgr/occupationalhealth/OHI_GuidanceManual_2018_FINA.pdf

[CR18] Dembe AE (2001). The social consequences of occupational injuries and illnesses. Am J Ind Med.

[CR19] Ellis GF, Lumley T, Schneider B, Greg M, Krivitsky PN. Package ‘srvyr’ (1.1.0). 2021.

[CR20] Fathallah F, Miller B, Miles J (2008). Low back disorders in agriculture and the role of stooped work: scope, potential interventions, and research needs. J Agric Saf Health.

[CR21] Gihleb R, Giuntella O, Stella L, Wang T (2022). Industrial robots, workers’ safety, and health. Labour Econ.

[CR22] Groenewold MR, Baron SL (2013). The proportion of work-related emergency department visits not expected to be paid by workers’ compensation: implications for occupational health surveillance, research, policy, and health equity. Health Serv Res.

[CR23] Guerin RJ, Reichard AA, Derk S, Hendricks KJ, Menger-Ogle LM, Okun AH (2020). Nonfatal occupational injuries to younger workers—United States, 2012–2018. MMWR Morb Mortal Wkly Rep.

[CR24] Hämäläinen P (2009). The effect of globalization on occupational accidents. Saf Sci.

[CR25] Hassi J, Gardner L, Hendricks S, Bell J (2000). Occupational injuries in the mining industry and their association with statewide cold ambient temperatures in the USA. Am J Ind Med.

[CR26] Ho V, Metcalfe L, Dark C, Vu L, Weber E, Shelton G (2017). Comparing utilization and costs of care in freestanding emergency departments, hospital emergency departments, and urgent care centers. Ann Emerg Med Am Coll Emerg Phys.

[CR27] Hyndman RJ, Athanasopoulos G. 8.9 Seasonal ARIMA models [Internet]. Forecasting: principles and practice. 2018a [cited 2022 Mar 19]. Available from: https://otexts.com/fpp2/seasonal-arima.html

[CR28] Hyndman RJ, Athanasopoulos G. 8.7 ARIMA modelling in R [Internet]. Forecasting: principles and practice. 2018b [cited 2022 Mar 19]. Available from: https://otexts.com/fpp2/arima-r.html

[CR29] Issa SF, Patrick K, Thomson S, Rein B (2019). Estimating the number of agricultural fatal injuries prevented by agricultural engineering developments in the United States. Safety.

[CR30] Janocha J, Hopler C (2018). The facts of the faller: occupational injuries, illnesses, and fatalities to loggers. Bureau Labor Stat [internet].

[CR31] Khairat S, Lin X, Liu S, Man Z, Zaman T, Edson B (2021). Evaluation of patient experience during virtual and in-person urgent care visits: time and cost analysis. J Patient Exp.

[CR32] Kim J, Choi Y (2016). Gender differences in the longitudinal association between work-related injury and depression. Int J Environ Res Public Health.

[CR33] Konda S, Reichard A, Tiesman HM, Hendricks S (2015). Non-fatal work-related traumatic brain injuries treated in US hospital emergency departments, 1998–2007. Inj Prev.

[CR34] Lax MB, Klein R (2008). More than meets the eye: social, economic, and emotional impacts of work-related injury and illness. New Solut J Environ Occup Health Policy.

[CR35] Leso V, Fontana L, Iavicoli I (2018). The occupational health and safety dimension of Industry 4.0. Med Lav.

[CR36] Lipscomb HJ, Schoenfisch AL, Shishlov KS, Myers DJ (2010). Nonfatal tool- or equipment-related injuries treated in US emergency departments among workers in the construction industry, 1998–2005. Am J Ind Med.

[CR37] Ljung GM, Box GEP (1978). On a measure of lack of fit in time series models. Biometrika [internet].

[CR38] Lumley T. Package ‘survey.’ 2021.

[CR39] Marsh SM, Reichard AA, Bhandari R, Tonozzi TR (2016). Using emergency department surveillance data to assess occupational injury and illness reporting by workers. Am J Ind Med.

[CR40] Monforton C, Windsor R (2010). an impact evaluation of a federal mine safety training regulation on injury rates among US stone, sand, and gravel mine workers: an interrupted time-series analysis. Am J Public Health [internet].

[CR41] Morse T, Punnett L, Warren N, Dillon C, Warren A (2003). The relationship of unions to prevalence and claim filing for work-related upper-extremity musculoskeletal disorders. Am J Ind Med.

[CR42] Murphy G, Patel J, Boden L, Wolf J. Workers’ compensation: benefits, costs, and coverage (2019 data) [Internet]. 2021 [cited 2022 Dec 20]. Available from: https://www.nasi.org/wp-content/uploads/2021/10/2021-Workers-Compensation-Report-2019-Data.pdf

[CR43] Myers JR, Kisner SM, Fosbroke DE (1998). Lifetime risk of fatal occupational injuries within industries, by occupation, gender, and race. Hum Ecol Risk Assess Int J.

[CR44] National Academy of Sciences. Current status of federal and state programs and cross-cutting issues. A smarter national surveillance system for occupational safety and health in the 21st century [Internet]. Washington, DC: National Academies Press; 2018 [cited 2023 Jan 29]. p. 73–142. Available from: https://nap.nationalacademies.org/catalog/24835/a-smarter-national-surveillance-system-for-occupational-safety-and-health-in-the-21st-century 29648768

[CR45] National Institute for Occupational Safety and Health (NIOSH) Division of safety research. Work-related injury statistics query system technical info [Internet]. 2021a [cited 2022 Dec 20]. Available from: https://wwwn.cdc.gov/Wisards/workrisqs/techinfo.aspx

[CR46] National Institute for Occupational Safety and Health (NIOSH) Division of Safety Research. Occupational injury and illness classification system [Internet]. 2021b [cited 2022 Dec 20]. Available from: https://wwwn.cdc.gov/wisards/oiics/

[CR47] National Institute for Occupational Safety and Health (NIOSH) Division of Safety Research. Employed labor force (ELF) query system technical info [Internet]. 2021c [cited 2022 Sep 15]. Available from: https://wwwn.cdc.gov/Wisards/cps/cps_techinfo.aspx#tic12

[CR48] National Institute for Occupational Safety and Health (NIOSH) Division of Safety Research. Rate calculations [Internet]. 2021d [cited 2022 Sep 15]. Available from: https://wwwn.cdc.gov/Wisards/workrisqs/rate.aspx

[CR49] National Institute for Occupational Safety and Health. Occupational violence [Internet]. 2022 [cited 2023 Jan 29]. Available from: https://www.cdc.gov/niosh/topics/violence/fastfacts.html

[CR50] National Research Council and Institute of Medicine Panel on Musculoskeletal Disorders and the Workplace. 8. Interventions in the Workplace. Musculoskeletal disorders and the workplace: low back and upper extremities. National Academy Press; 2001. pp. 301–27.

[CR51] National Safety Council. Contact with objects and equipment [Internet]. 2023 [cited 2023 Jan 29]. Available from: https://injuryfacts.nsc.org/work/safety-topics/contact-with-objects-and-equipment/

[CR52] National Safety Council. Work injury costs [Internet]. Injury Facts. [cited 2022 Dec 24] Available from: https://injuryfacts.nsc.org/work/costs/work-injury-costs/

[CR53] Oleske DM, Hahn JJ (1992). Work-related injuries of the hand: data from an occupational injury/illness surveillance system. J Community Health.

[CR54] Pierce B. The seasonal timing of work-related injuries October 2013. 2013. https://www.bls.gov/osmr/research-papers/2013/pdf/st130230.pdf

[CR55] Reichard A, Marsh S. Improving our understanding of nonfatal occupational injuries. NIOSH Science Blog. 2021. Available from: https://blogs.cdc.gov/niosh-science-blog/2021/04/13/neiss-work/

[CR56] Reichard AA, Konda S, Jackson LL (2015). Occupational burns treated in emergency departments. Am J Ind Med.

[CR57] Robinson AM, Smallman C (2006). The contemporary British workplace: a safer and healthier place?. Work Employ Soc.

[CR58] Rothwell J, Harlan J. Gig economy and self-employment report [Internet]. 2019. Available from: https://quickbooks.intuit.com/content/dam/intuit/quickbooks/Gig-Economy-Self-Employment-Report-2019.pdf

[CR59] Rstudio Team. Rstudio: integrated development for R [Internet]. Boston, MA: Rstudio, Inc.; 2022 [cited 2022 Mar 29]. Available from: http://www.rstudio.com/

[CR60] Schulze LJH. The impact of globalization on occupational safety and health. In: Proceedings of the ASSP 2007 Professional Development Conference and Exposition [Internet]. 2007 [cited 2022 Dec 24]. Available from: https://aeasseincludes.assp.org/proceedings/2007/docs/786.pdf

[CR61] Seabury SA, Scherer E, O’leary P, Ozonoff A, Boden L (2014). Using linked federal and state data to study the adequacy of workers’ compensation benefits. Am J Ind Med.

[CR62] Slavova S, Bunn TL (2015). Work-related concussion surveillance. Am J Ind Med.

[CR63] Taylor AJ, McGwin G, Valent F, Rue LW (2002). Fatal occupational electrocutions in the United States. Inj Prev.

[CR64] Tiesman HM, Gwilliam M, Konda S, Rojek J, Marsh S (2018). Nonfatal injuries to law enforcement officers: a rise in assaults. Am J Prev Med.

[CR65] Tiesman HM, Gwilliam M, Rojek J, Hendricks S, Montgomery B, Alpert G (2019). The impact of a crash prevention program in a large law enforcement agency. Am J Ind Med.

[CR66] U.S. Bureau of Labor Statistics. TABLE 1. Incidence rates of nonfatal occupational injuries and illnesses by industry and case types, 2012.[Internet]. Washington, D.C.; 2013 Nov. Available from: https://www.bls.gov/iif/nonfatal-injuries-and-illnesses-tables/soii-summary-historical/ostb3581.pdf

[CR67] U.S. Bureau of Labor Statistics. Union workers more likely than nonunion workers to have healthcare benefits in 2019 [Internet]. The Economics Daily. 2019 [cited 2022 Dec 20]. Available from: https://www.bls.gov/opub/ted/2019/union-workers-more-likely-than-nonunion-workers-to-have-healthcare-benefits-in-2019.htm

[CR68] U.S. Bureau of Labor Statistics. TABLE 2. Numbers of nonfatal occupational injuries and illnesses by industry and case types, 2019 [thousands] [Internet]. Injuries, Illnesses, and Fatalities. 2020a. Available from: https://www.bls.gov/iif/nonfatal-injuries-and-illnesses-tables/soii-summary-historical/summary-table-2-2019-national.htm

[CR69] U.S. Bureau of Labor Statistics. TABLE 1. Incidence rates of nonfatal occupational injuries and illnesses by industry and case types, 2019. [Internet]. Washington, D.C.; 2020b Nov. Available from: https://www.bls.gov/iif/nonfatal-injuries-and-illnesses-tables/soii-summary-historical/summary-table-1-2019-national.htm

[CR70] U.S. Bureau of Labor Statistics. HOUSEHOLD DATA Table A-9. Selected employment indicators [Internet]. Labor Force Statistics (CPS). 2020c [cited 2022 Dec 24]. Available from: https://www.bls.gov/webapps/legacy/cpsatab9.htm

[CR71] U.S. Bureau of Labor Statistics. All Employees, Manufacturing/All Employees, Total Nonfarm [Internet]. FRED, Federal Reserve Bank of St. Louis. 2022a [cited 2022a Dec 20]. Available from: https://fred.stlouisfed.org/graph/?g=cAYh#

[CR72] U.S. Bureau of Labor Statistics, All Employees, Health Care and Social Assistance [CES6562000001]. FRED, Federal Reserve Bank of St. Louis. 2022b [cited 2023 Jan 30]. Available from: https://fred.stlouisfed.org/series/CES6562000001

[CR73] Urgent Care Association. UCA 2019 Benchmarking Report. 2019. Available from: https://twitter.com/UrgentCareAssoc/status/1236049000593272838?lang=en

[CR74] Villaveces A, Mutter R, Owens PL, Barrett ML. STATISTICAL BRIEF #156: Causes of injuries treated in the emergency department, 2010 [Internet]. 2013 May. Available from: https://www.hcup-us.ahrq.gov/reports/statbriefs/sb156.pdf 24006548

[CR75] Wiatrowski WJ (2014). The BLS survey of occupational injuries and illnesses: a primer. Am J Ind Med.

[CR76] Wickizer TM, Kopjar B, Franklin G, Joesch J (2004). Do drug-free workplace programs prevent occupational injuries? Evidence from Washington State. Health Serv Res.

[CR77] Williams D. Survey nonresponse trends, challenges, and strategies. Washington, D.C.; 2022 Dec. Available from: https://apps.bea.gov/fesac/meetings/2022-12-09/williams-survey%20response.pdf

[CR78] Witt WS, Bunn TL, Slavova S (2018). Workers compensation-reported injuries among security and law enforcement personnel in the private versus public sectors. Inj Epidemiol.

[CR79] Wurzelbacher SJ, Meyers AR, Lampl MP, Timothy Bushnell P, Bertke SJ, Robins DC (2021). Workers’ compensation claim counts and rates by injury event/exposure among state-insured private employers in Ohio, 2007–2017. J Safety Res.

[CR80] Zhang X, Zhang T, Young AA, Li X (2014). Applications and comparisons of four time series models in epidemiological surveillance data. PLoS ONE.

[CR81] Zhu H, Wilson FA, Stimpson JP, Hilsenrath PE (2015). Rising gasoline prices increase new motorcycle sales and fatalities. Inj Epidemiol.

