# Supplementary figures and images for: Temporal trends in occupational injuries treated in US emergency departments, 2012–2019
Source: Inj Epidemiol. 2023 Mar 10;10:13. doi: 10.1186/s40621-023-00423-y (PMC9999541; doi:10.1186/s40621-023-00423-y)

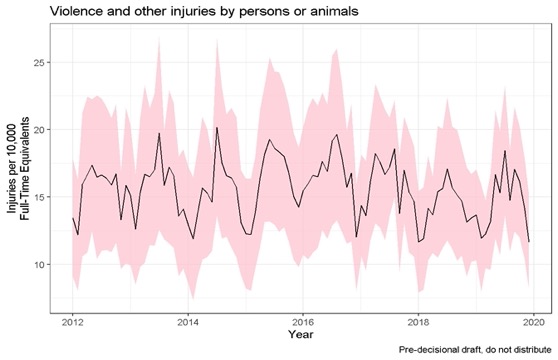

Supplement: Supplementary file 1 — Additional file 1: Fig. S1. Numerator data (monthly ED-treated injury count estimates associated with violence and other injuries by persons or animals) are from the National Emergency Injury Surveillance System—Occupational Supplement (NEISS-Work) dataset and were produced using the R packages “survey” and “srvyr.” Denominator data (FTE) were obtained from the Current Population Survey (CPS) via the NIOSH Employed Labor Force querying system. Variances from both numerator and denominator data were used to calculate for injury rate 95% CI using a Taylor series expansion. [file 40621_2023_423_MOESM1_ESM.docx]

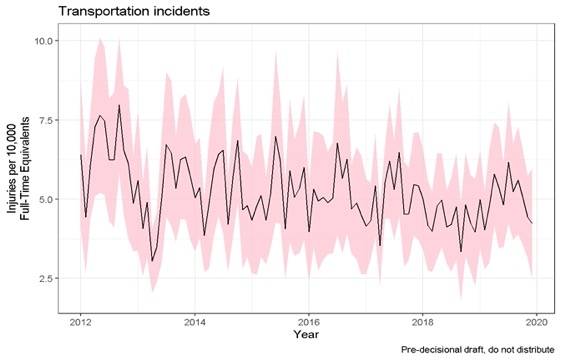

Supplement: Supplementary file 2 — Additional file 2: Fig. S2. Numerator data (monthly ED-treated transportation injury count estimates) are from the National Emergency Injury Surveillance System—Occupational Supplement (NEISS-Work) dataset and were produced using the R packages “survey” and “srvyr.” Denominator data (FTE) were obtained from the Current Population Survey (CPS) via the NIOSH Employed Labor Force querying system. Variances from both numerator and denominator data were used to calculate for injury rate 95% CI using a Taylor series expansion. [file 40621_2023_423_MOESM2_ESM.docx]

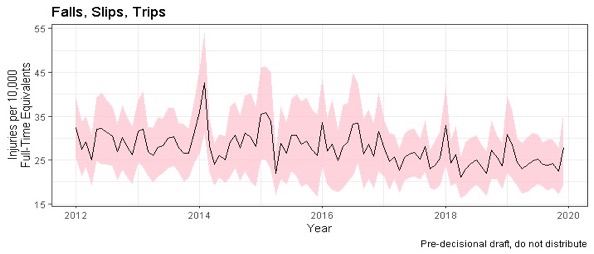

Supplement: Supplementary file 3 — Additional file 3: Fig. S3. Numerator data (monthly ED-treated falls, slips, and trips injury count estimates) are from the National Emergency Injury Surveillance System—Occupational Supplement (NEISS-Work) dataset and were produced using the R packages “survey” and “srvyr.” Denominator data (FTE) were obtained from the Current Population Survey (CPS) via the NIOSH Employed Labor Force querying system. Variances from both numerator and denominator data were used to calculate for injury rate 95% CI using a Taylor series expansion. [file 40621_2023_423_MOESM3_ESM.docx]

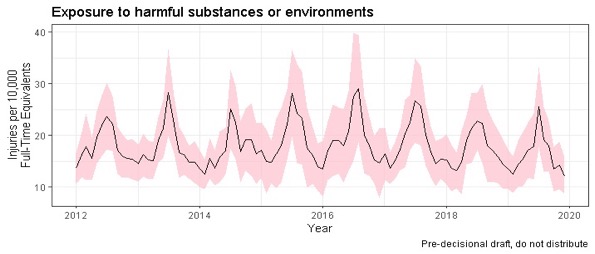

Supplement: Supplementary file 4 — Additional file 4: Fig. S4. Numerator data (monthly ED-treated injury count estimates associated with exposure to harmful substances or environments) are from the National Emergency Injury Surveillance System—Occupational Supplement (NEISS-Work) dataset and were produced using the R packages “survey” and “srvyr.” Denominator data (FTE) were obtained from the Current Population Survey (CPS) via the NIOSH Employed Labor Force querying system. Variances from both numerator and denominator data were used to calculate for injury rate 95% CI using a Taylor series expansion. [file 40621_2023_423_MOESM4_ESM.docx]

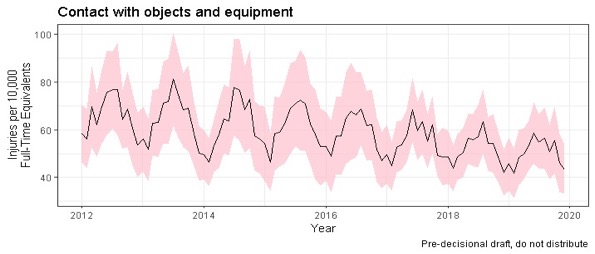

Supplement: Supplementary file 5 — Additional file 5: Fig. S5. Numerator data (monthly ED-treated injury count estimates associated with contact with objects and equipment) are from the National Emergency Injury Surveillance System—Occupational Supplement (NEISS-Work) dataset and were produced using the R packages “survey” and “srvyr.” Denominator data (FTE) were obtained from the Current Population Survey (CPS) via the NIOSH Employed Labor Force querying system. Variances from both numerator and denominator data were used to calculate for injury rate 95% CI using a Taylor series expansion. [file 40621_2023_423_MOESM5_ESM.docx]

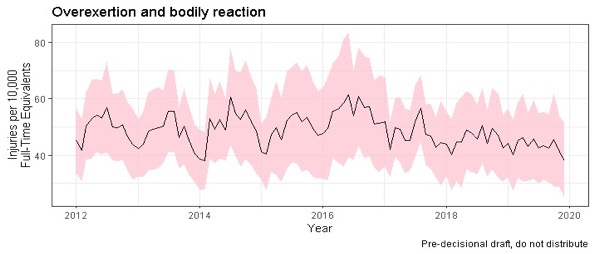

Supplement: Supplementary file 6 — Additional file 6: Fig. S6. Numerator data (monthly ED-treated injury count estimates associated with overexertion and other bodily reaction) are from the National Emergency Injury Surveillance System—Occupational Supplement (NEISS-Work) dataset and were produced using the R packages “survey” and “srvyr.” Denominator data (FTE) were obtained from the Current Population Survey (CPS) via the NIOSH Employed Labor Force querying system. Variances from both numerator and denominator data were used to calculate for injury rate 95% CI using a Taylor series expansion. [file 40621_2023_423_MOESM6_ESM.docx]
